# Backyard Livestock Guinea Pigs Are Reservoirs for *Leptospira* Shedding in Rural Households from Ecuador

**DOI:** 10.3390/tropicalmed10090256

**Published:** 2025-09-08

**Authors:** Mauricio Xavier Salas-Rueda, Fabricio Dugllay-Montaleza, Darwin Paredes-Núñez, Katherine Natalia Chávez Toledo, Angel Sebastián Rodríguez-Pazmiño, Elsy Carvajal, Maria Belen Paredes-Espinosa, Patricia Peralta-Ortiz, Jairo Guama-Tipas, Juan Pablo Garzon, Alexandra Narvaez, Solon Alberto Orlando, Miguel Angel Garcia-Bereguiain

**Affiliations:** 1 GLOBALGEN, Universidad Politécnica Salesiana, Calle Vieja 12-30 y Elia Liut, Cuenca 010105, Ecuador; 2 One Health Research Group, Universidad de Las Américas, Quito 170516, Ecuador; 3 Universidad Católica Santiago de Guayaquil, Guayaquil 090504, Ecuador; 4 Instituto Nacional de Investigaciones Agropecuarias (INIAP), Estación Experimental del Austro, Gualaceo 010350, Ecuador; 5 Universidad Espíritu Santo, Guayaquil 092301, Ecuador; 6 Instituto Nacional de Salud Pública e Investigación, Guayaquil 090514, Ecuador; 7 Universidad Ecotec, Guayaquil 092302, Ecuador

**Keywords:** guinea pigs, livestock, backyard, Ecuador, leptospirosis, *Leptospira*, MAT

## Abstract

Guinea pigs (*Cavia porcellus*) are bred as livestock in South American countries from the Andean region, including Ecuador. Despite their importance for the local rural economy, no public health management guidelines have ever been implemented for guinea pig farming. Moreover, several reports have shown the carriage of diverse zoonotic pathogens in livestock guinea pigs, including respiratory and enteric pathogens, or *Toxoplasma gondii*. Leptospirosis, a zoonotic disease caused by bacteria from *Leptospira* genus, is endemic in Ecuador and has multiple animal reservoirs, with rodents considered to be the main vector for transmission to humans. However, the role of livestock guinea pigs in the epidemiology of leptospirosis is poorly understood. In this study, the prevalence of antibodies against *Leptospira* in guinea pigs from Ecuador was analyzed with the Microaglutiantion Test (MAT). Moreover, the shedding of Leptospira by backyard livestock guinea pigs was also addressed, analyzing fecal and urine samples by qPCR for *lipL32*, *secY* and *rrs* genes. From the 118 guinea pigs analyzed by MAT, 61.9% were positive for 19 different pathogenic serovars, with Icterohaemorrhagiae, Pomona, Castellonis and Bataviae being the most prevalent ones. From the 231 urine samples and 225 fecal samples collected, 27.7% and 78.7% were positive for *Leptospira* by qPCR, respectively. Our results highlight the role of livestock guinea pigs as a reservoir for leptospirosis. Moreover, this study underscores the zoonotic threat associated with the shedding of *Leptospira* within households in rural communities where guinea pigs are bred as livestock. Animal and public health guidelines from a One Health perspective must be implemented to prevent leptospirosis transmission from guinea pigs in Ecuador and other countries in the Andean Region where the breeding of backyard livestock guinea pigs is common.

## 1. Introduction

The guinea pig (*Cavia porcellus*) is commonly used as a laboratory model or a pet in many Western countries [1]. However, it is also raised commonly as livestock in Andean countries of South America, including Colombia, Ecuador, Peru, and Bolivia, where its meat is part of the daily diet of millions of people [2]. Guinea pigs are still bred using very traditional methods. Although industrialized farms housing thousands of animals exist, guinea pigs are often raised in rural communities as backyard livestock in groups of up to 50 animals per household (see Supplementary Figure S1) [3]. In Ecuador alone, around 700,000 families are involved in guinea pig farming, with an estimated annual production of 47 million animals [4,5]. No specific management guidelines or official census for guinea pig farming have ever been implemented by animal or public health authorities to ensure food safety and quality [6]. In this sense, there is limited information on zoonotic infections associated with livestock guinea pigs [6]. The few studies available have reported the presence of respiratory pathogens [7,8,9,10], enteric pathogens including bacteria like *Campylobacter jejuni* [11] or parasites like *Blastocystis*, *Entamoeba* and *Cryptosporidium* [12]. Guinea pigs are also a reservoir for *Toxoplasma gondii* [13,14].

Leptospirosis is a zoonotic disease caused by the Gram-negative spirochete bacteria, *Leptospira* [15,16,17]. The taxonomy has been updated in recent years, based on the grounds of genomic information, dividing the genus *Leptospira* into more than 70 species, grouped into two main clades, P and S; each one of these clades has been divided into two subclades: subclade P1 (formerly described as the pathogen group), P2 (formerly described as the intermediate group), S1 and S2 (formerly described as the saprophyte group) [18]. Leptospirosis diagnosis is based in serological methods like the Microagglutination Test (MAT) that allow the identification of different serogroups and serovars within each species, and it is still considered to be the gold standard [19,20,21]. But also, PCR has been included as a confirmatory diagnosis tool, with several gene targets like *lipL32*, *secY* or *rrs* recommended for identification of P1 and P2 sublcades of pathogenic *Leptospira* [22,23]. Although rodents have been traditionally considered to be the main reservoir for zoonotic transmission to humans, the extensive diversity of the *Leptospira* species allows it to infect a wide range of both wild and domestic animals including dogs, pigs and cows [19,20,21,22]. These animal reservoirs can spread the bacteria into the environment through their urine, so the principal transmission pathway includes direct contact with contaminated water, food or fluids containing animal urine that is infected with *Leptospira* [24]. Although *Leptospira* cannot replicate outside of the host, it could remain viable for up to 180 days in water and soil, especially under tropical climate conditions, making leptospirosis endemic to the tropics [25,26,27]. Leptospirosis causes significant morbidity and mortality in humans in Ecuador [28], and several studies carried out in recent years have already shown a high prevalence of *Leptospira* infections and diversity of pathogenic species and serovars in multiple animal reservoirs, like dogs, horses, cattle, pigs, wild mammals or rats [16,19,20,21,22,24,29,30,31,32,33,34,35,36].

Rodents are considered to be the main reservoirs for leptospirosis outbreaks [22]. However, the role of guinea pigs in the epidemiology of leptospirosis in the Andean Region is poorly studied. There are very few preliminary reports from Colombia [36], Ecuador [37] and Peru [38,39] where the presence of positive guinea pigs by a reduced panel of serovars (MAT) or conventional PCR is described. However, these studies were carried out with guinea pigs from commercial facilities and low-sensitivity diagnostic tools were used.

In this sense, the aim of this work was to study the seroprevalence and diversity of *Leptospira* pathogenic serovars in backyard livestock guinea pigs from Ecuador. Moreover, the shedding of *Leptospira* from guinea pigs within households from rural communities in Ecuador was also addressed.

## 2. Materials and Methods

### 2.1. Study Setting

The study was carried out in Azuay, a province in Ecuador located in the Andean region at an elevation of 2500 m above sea level and one of the leading producers of livestock guinea pigs in the country. This cross-sectional study was carried out in two phases:

Phase I: Blood samples from 118 guinea pigs were collected at slaughterhouses in Cuenca canton (Azuay province) throughout 2022. Blood samples were obtained from healthy guinea pigs, selected based on convenience sampling at slaughterhouses, depending on the availability of these facilities to permit sample collection. There were no exclusion criteria, and any animal slaughtered while we were present was included in the study. Although the convenience sampling method has the limitation of potential selection bias, it was the only possible approach to carry out this study, as guinea pig breeders were not willing to allow blood sample collection outside of the slaughterhouses. All the animals included in the study were healthy individuals in the range of 3–5 months old, and were ready to be sold for human consumption

Phase II: Urine and fecal samples were collected from backyard livestock guinea pigs in Cuenca canton (Azuay province) throughout 2023. By backyard livestock guinea pigs, we mean guinea pigs that are bred in small farms (less than 50 animals) for self-consumption within the household in rural communities with very poor sanitary conditions and a lack of veterinary counseling (Supplementary Figure S1). Because of the lack of an official census of guinea pig farms in Ecuador, the inclusion of farms in this study was performed at convenience, following a “snowball” approach to contact and recruit neighbor guinea pig breeders. All the animals included in the study were healthy individuals in the range of 3–5 months old, ready to be sold for human consumption. Access to older animals (reproductive males and females) was not granted by farmers. Nine farms were included in the study and overall, 223 fecal samples and 231 urine samples were collected. When possible, both urine and feces samples were collected from each animal, although not all the samples for urine/feces were paired in all the animals (sometimes it was not possible to collect urine; other times, the fecal sample was not of good quality for DNA extraction).

### 2.2. Blood Sample Collection

Two mL of blood were collected from the jugular vein using red-top tubes containing serum clot activators. Veterinary staff carrying protective gear and sterile material were used for sample collection to guarantee an aseptic blood extraction. The samples were stored at 4 °C (ice bucket with thermometer to control temperature) within 1 min after collection and transported to the laboratory within 2 h after collection. After clotting, 1 to 1.5 mL of serum was separated and transferred into 2 mL Eppendorf tubes. Serum samples were stored at −20 °C until analysis within 5 h after collection.

### 2.3. Fecal and Urine Sample Collection

Fecal samples were collected from the guinea pig’s rectum with a sterile rayon swab. Veterinary staff carrying protective gear and sterile material were used for the sample collection to guarantee an aseptic sample collection. The swab was placed within 0.5 mL of Tris-EDTA buffer and stored at 4 °C (in an ice bucket with thermometer to control temperature) within 1 min after collection and transported to the laboratory within 2 h after collection. Fecal samples were stored at −20 °C until analysis within 5 h after collection.

Urine samples were obtained from guinea pigs by cystocentesis, using a sterile needle and syringe, with the animals properly restrained to minimize stress. Approximately 2 mL of urine was collected per individual, ensuring sample integrity and avoiding contamination, and samples were also kept at 4 °C until processing in the laboratory. Samples were stored at −20 °C until analysis within 5 h after collection.

### 2.4. Microscopic Agglutination Test for Detection of Antibodies Against Leptospirosis

The Microscopic Agglutination Test (MAT) was performed using 24 live antigens on a panel. For details of *Lesptospira’s* serogroups, serovars and strains of the MAT panel, see Supplementary Table S1. The antigens were prepared from the reference strains included in the panel. For the screening of sera, a 1:200 elution was the starting one used and the cut off. Reactive samples were then examined with increasing dilutions from 1:200 to 1:3200, taking the highest positive elution to be the titer of the serum. The serum was taken as reactive when at least 50% of agglutination occurred at 40× under the microscope. To address coagglutination in the MAT assay, the number of positive serovars for each animal was also calculated. When a serum specimen reacted with two or more serovars, we considered equally dominant serovars to those serovars with the higher title. However, for the calculation of the overall prevalence of every single serovar, we considered all the serum specimens that reacted to this serovar, no matter if they were also positive for other serovars with a higher titer.

### 2.5. DNA Extraction and Identification of Leptospira by qPCR

DNA extraction for all samples was then carried out using a column method with the Purelink Genomic DNA kit (Invitrogen, Waltman, MA, USA), following the manufacturer’s instructions. For sample processing, 200 µL of either fecal or urine samples were used for DNA extraction.

For the detection of *Leptospira*, real-time PCR (qPCR) was employed with specific primers and probes targeting *lipL32*, *secY* and *rrs* genes, and internal control to monitor the presence of potential amplification inhibitors (*b-actin* gene). Primers and probe sequences used for the *lipL32* gene, included the forward primer (F_lip32: AAG CAT TAC CGC TTG TGG TG), reverse primer (R_lip32: GAA CTC CCA TTT CAG CGA TT), and probe (taq-189P: FAM-AAA GCC AGG ACA AGC GCC G-BHQ1). For the *secY* gene, the forward primer (F_Lint2: CTT GAG CCT GCG CGT TAY C), reverse primer (R_Lint2: CCG ATA ATT CCA GCG AAG ATC) and probe (TaqLint2: HEX-CTC ATT TGG TTA GGA GAA CAG ATC A-BHQ1) were used. For the *rrs* (16S) gene, the forward primer (F_Lept: CCCGCGTCCGATTAG), reverse primer (R_Lept: TCCATTGTGGCCGRA/GACAC) and probe (P_Lept: FAM-CTCACCAAGGCGACGATCGGTAGC-BHQ1) were utilized. Finally, for the *b-actin* gene, the forward primer (F_actin: GGC TCY ATY CTG GCC TC), reverse primer (R_actin: GCA YTT GCG GTG SAC RAT G) and probe (P_actin: Cy5-TAC TCC TGC TTG CTG ATC CAC ATC-BHQ2) were employed [22]. The qPCR reaction mixture comprised 2× Universal Master Mix primers at a final concentration of 0.2 μM, probes at a final concentration of 0.13 μM and nuclease-free water. Each well received 5 μL of DNA, added to 15 μL of Master Mix. qPCRs were executed on a CFX-96 real-time thermal cycler (BioRad, Hercules, CA, USA), employing the following conditions: initial denaturation at 95 °C for 2 min, followed by 45 cycles of denaturation at 95 °C for 15 s, and annealing/extension at 60 °C for 1 min. Positive and non-template controls were included in all runs. A Ct value of 40 was set as a threshold for positivity for all the genes [22].

### 2.6. Statistical Analysis

The data were processed and analyzed using EpiInfo version 7.2.5.0. Prevalence percentages, along with 95% confidence intervals (Wilson score method, as per software default settings), were calculated. The chi-square test was used to compare prevalence among serovars and the positivity rate for urine/fecal samples by qPCR. A *p*-value of <0.05 was considered statistically significant.

### 2.7. Ethics Statement

According to Ecuadorian national regulations, IRB approval is not required for sample collection for diagnosis in farm animals. Animal handling and sample collection were conducted by veterinarians in accordance with animal welfare standard protocols. Informed consent was collected from guinea pig breeders, who supervised sample collection.

## 3. Results

### 3.1. Seroprevalence of Antibodies Against Leptospira and Serovars Distribution in Backyard Livestock Guinea Pigs

The overall prevalence of antibodies against *Leptospira* was 61.9% (CI 95% 52.5–70.7). The titer range obtained for MAT was 1/200 to 1/1200 depending on the serovar (Table 1). Moreover, 31 samples were positive multiple serovars (two to eight different serovars) and coagglutination was very frequent due to cross-reactivity between the different serovars.

There were positive guinea pigs for 19 pathogenic serovars out of the 24 ones tested (Table 1). The most prevalent serovars were Icterohaemorrhagiae (19.5%; CI 95%: 12.8–27.8), Pomona (16.9%; CI 95%: 10.7–25.0), Castellonis (11.9%; CI 95%: 6.6–19.1) and Bataviae (10.2%; CI 95%: 5.4–17.1). The differences in prevalence of serovars were statistically significant (*p* < 0.0001).

### 3.2. Leptospira Excretion by Backyard Livestock Guinea Pigs Assessed by qPCR

Table 2 shows the detection of *Leptospira* in urine and fecal samples from guinea pigs. Out of 231 urine samples, 27.7% (CI 95%: 22.1–34.0) were positive for *Leptospira.* The average values for the Ct obtained for urine samples were 36.7, 32.8 y 31.2 for the genes *lipL32* (range: 35.4–37.9; n = 2), *rrs* (range: 27.6–37.9; n = 57) and *secY* (range: 24.6–37.8; n = 41), respectively. 

Out of 225 fecal samples tested, 78.7% (CI 95%: 72.7–83.8) were positive for *Leptospira*. The average values for the Ct obtained for fecal samples were 30.8 (range: 23.6–37.9; n = 177) for *rrs* and 27.9 (range: 24.3–31.5; n = 29) for *secY.* There was a single positive sample for *lipL32* with Ct of 36.4.

The differences in the overall positivity rate for *Leptospira* by qPCR for fecal and urine samples were statistically significant (*p* < 0.001).

Table 3 shows the detection of *Leptospira* in urine and fecal samples from guinea pigs for each of the nine farms included in the study. For fecal samples, farm nine had no *Leptospira* positive animals, while farm two had the highest prevalence (20.9%); those differences between farms were statistically significant (*p* < 0.0001). For urine samples, farms one, three, five and eight had no *Leptospira* positive animals, while farm four had the highest prevalence (48.4%); those differences between farms were statistically significant (*p* < 0.0001). Overall, the nine samples were positive for *Leptospira* either in fecal, urine or both types of samples.

## 4. Discussion

Our study shows a high prevalence of over 60% of antibodies against *Leptospira* and a wide diversity of 19 pathogenic serovars in livestock guinea pigs from the Azuay province of Ecuador. The serovars Icterohaemorrhagiae, Pomona, Castellonis and Bataviae were the most prevalent ones, with values over 10%. Additionally, a high prevalence of *Leptospira* infection was found in backyard livestock guinea pigs from rural communities, with positivity rates by qPCR of 27.7% in urine and 78.7% in feces, underscoring the role of guinea pigs as spreaders of leptospirosis within rural households in Ecuador.

There were several reports performed in Ecuador in the last 10 years addressing the epidemiology of leptospirosis where a high prevalence of infection in a wide diversity of animal reservoirs has been described, including wild fauna, rats or domestic animals like dogs, cats, horses, cows and pigs [19,20,21,22,23,24,25,26,27,28,29,30,31,32,33,34,35,40,41]. However, despite the importance of guinea pigs for Ecuadorian livestock production, there is only a single study reporting *Leptospira* in these animals [37]. However, that study was performed only with commercial farms, using a reduced MAT panel of only six serovars and a low-sensitivity PCR protocol for diagnosis; despite those limitations, the infection with *Leptospira* was detected at a low prevalence [37]. Additionally, there were also three studies performed in Peru using MAT where prevalence values for antibodies against *Leptospira* ranging from 30 to 40% were reported [38,39,42]. There is also a single study performed in Colombia [36], with the same limitations described for the study in Ecuador, where the infection with *Leptospira* was detected at a low prevalence. In our study, we used sensitive diagnostic methods (24 serovars MAT panel and qPCR) to confirm the high prevalence of antibodies against *Leptospira* and a wide diversity of pathogenic serovars, but also the shedding of *Leptospira* within households of rural communities in Ecuador, where backyard livestock guinea pigs are bred. Interestingly, a higher shedding was found in feces than in urine from guinea pigs in our study. However, the most plausible explanation for that finding is the ingestion of forage contaminated with *Leptospira* coming from the urine of the guinea pigs, as they are kept in large groups and low sanitary conditions within their breeding cages (see Supplementary Figure S1). As it has been described for other rodents, *Leptospira* shedding by guinea pigs would be associated with urine excretion [22,24]. Our study contributes to the complex epidemiology of leptospirosis in Ecuador by adding another important animal reservoir that could contribute to the circulation of *Leptospira* and eventually transmission to humans. In this sense, the most prevalent serovars found in guinea pigs in the current study (Pomona, Icterohaemorrhagiae, Castellonis and Bataviae) have also been described as highly prevalent in rats, dogs, cats, pigs and cows in Ecuador [19,20,21].

Our lab and other colleagues from the Andean Region have previously reported several zoonotic threats associated with guinea pigs in Ecuador, including respiratory and enteric pathogens, or *Toxopasma gondii* [7,8,9,10,11,12,13,14]. In this study, we have contributed to the field of zoonotic diseases associated with guinea pigs underscoring their role in the epidemiology of leptospirosis, a neglected zoonosis disease endemic to Ecuador on other countries in the Andean Region, where seasonal outbreaks affecting humans are frequent and lethal [22].

This study has some limitations that we would like to disclose. First, we carried out a single MAT for every guinea pig sample, so we could not address antibodies titer increases, as it is recommended to confirm active infection with *Leptospira* by this serological method; in this sense, our MAT results must be interpreted as the cumulative exposure of guinea pigs to *Leptospira*. Nevertheless, active infection was confirmed by qPCR in the backyard livestock guinea pig study group. Second, as multiple serovars were found with similar titer in the same guinea pig, coagglutination should be considered and the interpretation of results related to serovars distribution should be taken with caution (Coagglutination is frequent in MAT due to cross-reactivity between different serovars). Finally, we attempted Sanger sequencing of positive samples for *Leptospira* by qPCR, but the quality of the sequences obtained was too poor to allow any phylogenetic analysis. So, further studies are needed to characterize the species of *Leptospira* shed by guinea pigs.

In conclusion, our study highlights that livestock guinea pigs are reservoirs for multiple pathogenic serovars of *Leptospira*, and that this pathogen is actually spread by backyard guinea pigs bred within the households of rural communities in Ecuador. Those findings underscore the need for further research with a One Health perspective to develop public health guidelines for hygienic and safe guinea pig farming. This is particularly relevant in the Andean Region of the Americas where guinea pigs are consumed by millions of people daily.

## Figures and Tables

**Table 1:** Distribution and titers for serovars of *Leptospira* by MAT in sera from backyard livestock guinea pigs raised of Ecuador. Only serovars with positive titers are included.

Serogroups	Titer Range	n	%	CI 95%
Icterohaemorrhagiae	1/200–1/400	23	19.5	12.8–27.8
Pomona	1/200–1/1200	20	16.9	10.7–25.0
Castellonis	1/200–1/400	14	11.9	6.6–19.1
Bataviae	1/200–1/400	12	10.2	5.4–17.1
Patoc	1/200–1/400	10	8.5	4.1–15.1
Wolffi	1/200–1/800	8	6.8	3.0–12.9
Autumnalis	1/200–1/800	6	5.1	1.9–10.7
Grypothyphosa	1/200	6	5.1	1.9–10.7
Shermani	1/200–1/800	3	2.5	0.5–7.3
Saxkoeby	1/200–1/800	3	2.5	0.5–7.3
Panama	1/200	3	2.5	0.5–7.3
Andamana	1/200–1/400	3	2.5	0.5–7.3
Cynopteri	1/400	2	1.7	0.2–6.0
Hebdomadis	1/200	2	1.7	0.2–6.0
Brastilava	1/200	2	1.7	0.2–6.0
Sejroe	1/400	2	1.7	0.2–6.0
Tarassovi	1/400	1	0.8	0.1–4.6
Canicola	1/400	1	0.8	0.1–4.6
Djasiman	1/800	1	0.8	0.1–4.6

**Table 2:** Detection of *Leptospira* in urine and fecal samples of guinea pigs by qPCR for gene targets *lipL32*, *secY* and *rrs* (ratio means positive samples/total samples).

Sample Type	Total Sample Size	Overall qPCR Positivity (%)/Ratio/CI 95%	*lipL32* Positivity (%)/Ratio/CI 95%	*secY* Positivity (%)/Ratio/CI 95%	*rrs* Positivity (%)/Ratio/CI 95%
Urine	231	27.7 (64/231) (22.1–34.0)	0.9 (2/231) (0.11–3.1)	17.8 (41/231) (13.1–23.3)	24.7 (57/231) (19.3–30.8)
Feces	225	78.7 (177/225) (72.7–83.8)	0.4 (1/225) (0.01–2.5)	12.9 (29/225) (8.8–18.0)	78.7 (177/225) (72.7–83.8)

**Table 3:** Detection of *Leptospira* in urine and fecal samples of guinea pigs by qPCR in each individual farm.

Guinea Pig Farm	Total Fecal Samples	Positive Fecal Samples	Fecal qPCR Positivity (%) (CI 95%)	Total Urine Samples	Positive Urine Samples	Urine qPCR Positivity (%) (CI 95%)
Farm 1	20	15	8.4 (4.8–13.5)	13	0	0.0 (0.0–5.6)
Farm 2	50	37	20.9 (15.1–27.6)	20	5	7.8 (2.5–17.3)
Farm 3	20	20	11.3 (7.0–16.9)	20	0	0.0 (0.0–5.6)
Farm 4	20	17	9.6 (5.7–14.9)	42	31	48.4 (35.7–61.2)
Farm 5	20	20	11.3 (7.0–16.9)	37	0	0.0 (0.0–5.6)
Farm 6	31	25	14.1 (9.3–20.1)	20	10	15.6 (7.7–26.8)
Farm 7	41	38	21.4 (15.6–28.2)	21	1	1.5 (0.0–8.4)
Farm 8	10	5	2.8 (0.9–6.4)	10	0	0.0 (0.0–5.6)
Farm 9	13	0	0.0 (0.0–2.0)	48	17	26.5 (16.3–39.0)
Total samples	225	177	78.7 (72.7–83.8)	231	64	27.7 (22.1–34.0)

## Data Availability

Data is included in the main figures and tables of the manuscript, or in the Supplementary Materials. Any further details would be provided by authors upon request.

## Supplementary Materials

The following supporting information can be downloaded at: https://www.mdpi.com/article/10.3390/tropicalmed10090256/s1, Figure S1: Pictures showing a traditional backyard livestock guinea pigs breeding facility in Ecuador; Table S1: Panel of 24 reference strains for multiple serovars of *Leptospira* used for the MAT in this study.
