# Supplementary material for: Backyard Livestock Guinea Pigs Are Reservoirs for Leptospira Shedding in Rural Households from Ecuador
Source: Trop Med Infect Dis. 2025 Sep 8;10(9):256. doi: 10.3390/tropicalmed10090256 (PMC12474318; doi:10.3390/tropicalmed10090256)

Supplementary Table S1. Panel of 24 reference strains for multiple serovars of *Leptospira* used for the MAT in this study.

N°	SPECIES	SEROGROUP	SEROVAR	STRAIN
	**Pathogenic**			
**1**	*L. santarosai*	Shermani	Shermani	1342 k
**2**	*L. interrogans*	Bataviae	Bataviae	Van tienen
**3**	*L. kirschneri*	Cynopteri	Cynopteri	3522c
**4**	*L. interrogans*	Hebdomadis	Hebdomadis	Hebdomadis
**5**	*L. borgpetersenii*	Tasassovi	Tasassovi	Perepelitsin
**6**	*L. interrogans*	Icterohaemorrhagiae	Icterohaemorrhagiae	Ictero I
**7**	*L. borgpetersenii*	Sejroe	Saxkoebing	Mus 24
**8**	*L. interrogans*	Sejroe	Hardjo	Hardjoprajtino
**9**	*L. interrogans*	Pomona	Pomona	Pomona
**10**	*L. interrogans*	Sejroe	Wolffi	3705
**11**	*L. interrogans*	Autumnalis	Autumnalis	Akiyami A
**12**	*L. interrogans*	Canicola	Canicola	Hond Utrecht IV
**13**	*L. weilii*	Celledoni	Celledoni	Celledoni
**14**	*L. kirschneri*	Grippotyphosa	Grippotyphosa	Moska V
**15**	*L. interrogans*	Pyrogenes	Pyrogenes	Salinem
**16**	*L. interrogans*	Australis	Bratislava	Jez Bratislava
**17**	*L. santarosai*	Hebdomadis	Borincana	HS 622
**18**	*L. noguchii*	Panama	Panama	CZ 214
**19**	*L. interrogans*	Icterohaemorrhagiae	Copenhageni	M20
**20**	*L. borgpetersenii*	Ballum	Castellonis	Castellon 3
**21**	*L. borgpetersenii*	Javanica	Javanica	Veldrat Batavia 46
**22**	*L. interrogans*	Australis	Australis	Ballico
**23**	*L. borgpetersenii*	Sejroe	Sejroe	M 84
**24**	*L. interrogans*	Djasiman	Djasiman	Djasiman

Supplementary Figure S1. Pictures showing a traditional backyard livestock guinea pigs breeding facility in Ecuador.

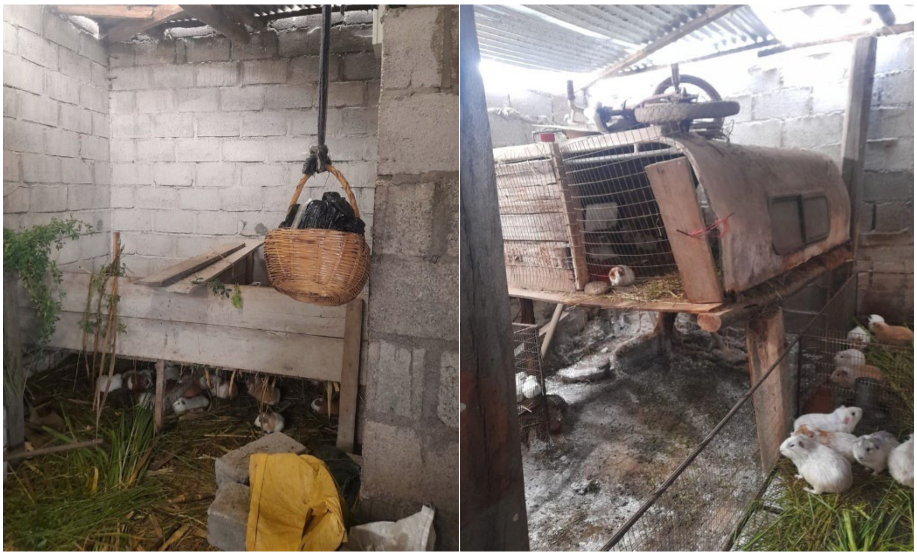

Supplement: Supplementary file 1 [file tropicalmed-10-00256-s001.zip › tropicalmed-3770415-supplementary.pdf]
